# Change of Cervical Cancer Stage at Diagnosis After the COVID-19 Pandemic

**DOI:** 10.7759/cureus.104659

**Published:** 2026-03-04

**Authors:** Fabian Tobon-Osornio, Abril A Arellano-Llamas, Paola M Perales-Flores, Carlos A Torrecilla-Toledo, Luis U Martínez-Chávez, Ana L Sandoval-Mejía, Alvaro Hernández-Caballero, Juan L Aboites-Lucero

**Affiliations:** 1 Surgical Oncology, Hospital de Gineco Obstetricia No. 3 "La Raza" Instituto Mexicano del Seguro Social, Mexico City, MEX; 2 Clinical Research, Hospital de Gineco Obstetricia No. 3 "La Raza" Instituto Mexicano del Seguro Social, Mexico City, MEX; 3 Pathology, Hospital de Gineco Obstetricia No. 3 "La Raza" Instituto Mexicano del Seguro Social, Mexico City, MEX; 4 División de Evaluación de Material de Curación y Dispositivos Médicos, Instituto Mexicano del Seguro Social, Mexico City, MEX

**Keywords:** cancer cervix screening, covid-19 pandemic, early detection of cancer, neoplasm staging, uterine cervical neoplasms

## Abstract

Background

Cervical cancer stage determines prognosis. Screening programs aim to detect cancer early, but the COVID-19 pandemic reduced cervical screening in Mexico by 79%. Our objective was to evaluate changes in the clinical stage at diagnosis, histologic type, time from screening to treatment, and time from diagnosis to treatment of patients with cervical cancer diagnosed before (2019), and during the pandemic (2020-2023).

Methods

Retrospective secondary data analysis of all newly diagnosed cervical cancer cases at a reference center from 2019 to 2023. Stages at diagnosis before, during, and after the COVID-19 pandemic were compared using the Chi-squared test, time from diagnosis to treatment was tested by the Mann-Whitney U Test.

Results

Compared to the pre-pandemic period, there was a significant change in the number of patients with cervical cancer during the pandemic period: -19.48% in 2020, followed by a maximum increase of +36% in 2022. Early-stage cervical cancer cases changed by -16.3%, -44.3%, -11.8%, and +6.4% during 2020-2023; locally advanced stages changed by -7.4%, +6.5%, +10%, and -6.4%; and metastatic cases by +94.4%, +58.2%, +36.6%, and +48.4%. Epidermoid cases decreased by -15%, -17%, -11%, and -4%; and adenocarcinoma increased by +72%, +78%, +52%, and +20% in the studied period. Time from screening to treatment increased by 2021 but returned to pre-pandemic lapse in 2023.

Conclusion

Early-stage cervical cancer decreased during the COVID-19 pandemic, while metastatic stages increased significantly, along with a significant change in histological types, time from screening to diagnosis increased since the COVID-19 pandemic.

## Introduction

The COVID-19 pandemic has severely disrupted the health systems of many countries, including Mexico. Health facilities were overwhelmed. Many hospitals and primary care offices were forced to operate beyond capacity. 

The Mexican Institute of Social Security (IMSS) is the largest healthcare provider in Mexico. It grants healthcare to more than sixty million Mexicans. The Gynecology and Obstetrics Hospital of La Raza Medical Center (HGOCMR) is one of the most relevant tertiary centers for the treatment of gynecological cancers in Mexico. It serves approximately 4,571,000 women aged 20 to 59. During the COVID-19 pandemic, IMSS repurposed 184 hospitals to screen and treat patients with respiratory diseases. Regardless, the HGOCMR continued to deliver maternal and oncology care, including cervical cancer treatment.

One of the most disrupted medical services during the COVID-19 pandemic was preventive care, including cervical cancer detection. An interrupted time-series analysis of health information system data in Mexico reported a 79% decrease in women screened for cervical cancer. Screening numbers dropped from 216,808 per month (SD 15,826) before COVID to 84,752 (SD 26,005) during the first wave of the emergency [1]. This decrease may be linked to the popular belief that people could become infected if they left their homes. It may also be due to the government's strict isolation policies in place for several weeks. 

The effect of the lockdown and the decrease in use of screening services for cervical cancer could have an impact on the time needed to detect and treat this neoplasm. Therefore, it may have changed the distribution of clinical stages at diagnosis in patients attended in the early phases of the pandemic compared to the late pandemic period.

A study compared the number of Papanicolaou (Pap) tests performed before the SARS-CoV-2 pandemic with those performed two years after the virus appeared in Brazil. It reported that the proportion of positive cervical intraepithelial neoplasia (CIN) cases increased significantly [2]. An increase in women with precancerous cervical lesions may produce a change in the proportion of clinical stages at diagnosis over time. This could cause a rise in advanced-stage occurrences, as cancer progression takes time [3].

The reduction in regular cervical cancer screening during the COVID-19 pandemic is likely to have caused delays in diagnosis. Such delays may affect both the allocation of health system resources and the outcomes for cervical cancer patients [4]. 

The objective of this study was to evaluate, from a real-world perspective, the changes in the clinical stage at diagnosis among cervical cancer patients and to measure the time from screening to treatment (TST) and the time from diagnosis to treatment (TDT) in a tertiary medical center in Mexico during the COVID-19 pandemic.

## Materials and methods

We conducted a retrospective analysis of all the medical records of cervical cancer cases diagnosed from 2019 to 2023. The FIGO Gynecologic Oncology Committee revised the staging of cancer of the cervix uteri in 2019 [5]. To accurately compare the staging of all patients in this study, all clinical stages were reclassified prospectively by a certified oncologist using the 2019 FIGO staging system, based on the clinical and radiological data reported in the clinical files. 

We recorded the interval from Pap test to first treatment, including palliative care for those ineligible for active treatment. TDT was calculated from the histopathology report date to initial treatment, and TSD from the last Pap test to the first treatment. Treatment types were categorized as surgery (with or without chemotherapy or radiotherapy) or radiotherapy (with or without other treatments except surgery). Obstetric history, comorbidities, and BMI (calculated and categorized by WHO criteria) were extracted from medical files. The pre-COVID-19 period was January-December 2019; the early pandemic, January 2020-December 2021; and the late pandemic, January 2022-December 2023. 

We used frequencies and percentages to describe categorical variables. For quantitative variables, we analyzed the distribution using the Kolmogorov-Smirnov test. If a normal distribution was present, we reported the mean and standard deviation. Otherwise, we reported the median and interquartile range. We used the chi-square test to compare the proportions of clinical stage at diagnosis across each pandemic period. Time lapses and BMI were compared by the Kruskal-Wallis test and the Mann-Whitney U test. The analysis was performed using SPSS V25 (IBM Inc., Armonk, New York).

The study was approved by an Ethical and Research Committee, with registration numbers R-2024-3504-018 and R-2025-3504-030.

## Results

**Figure 1:**
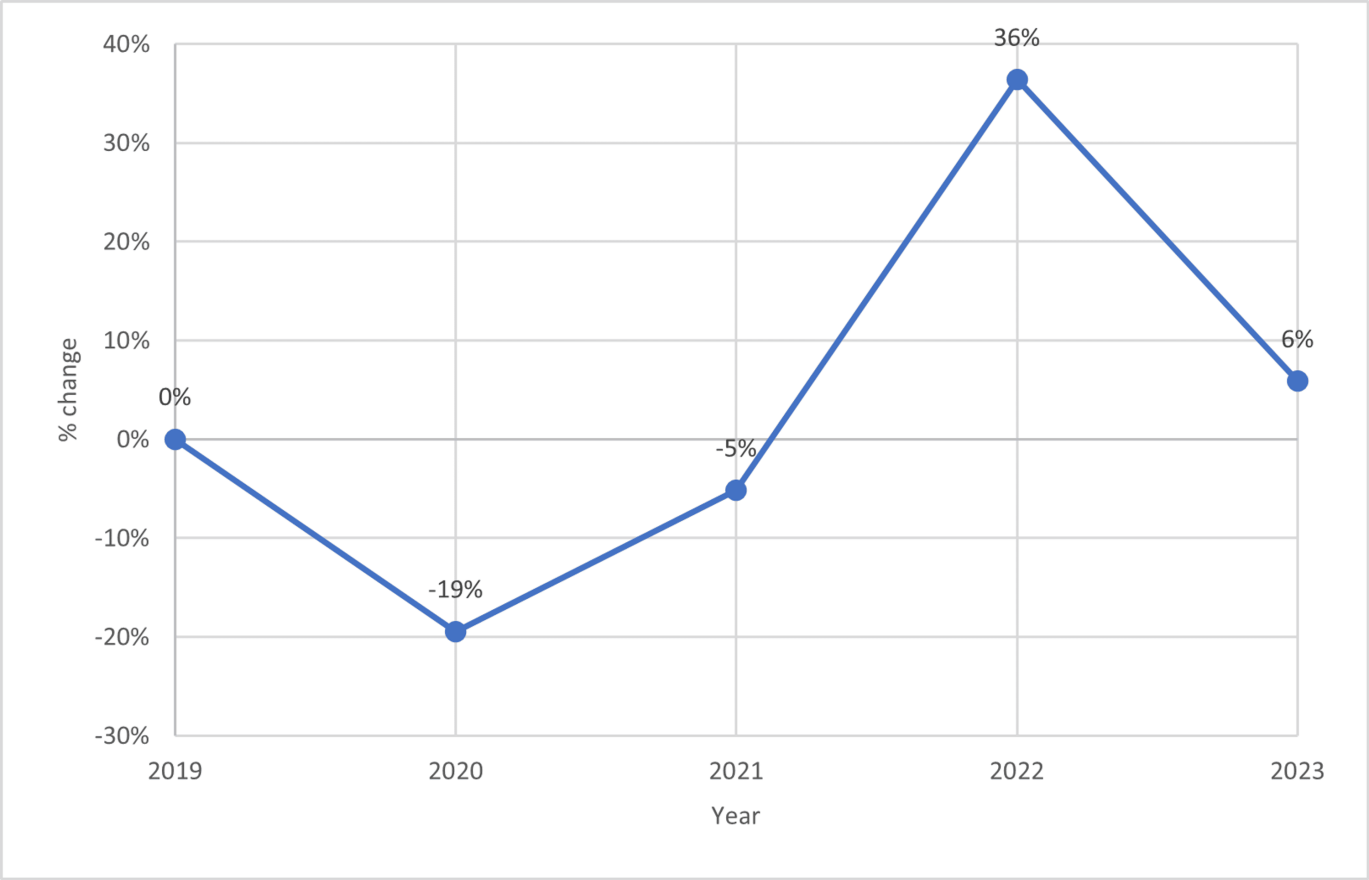
Change of cervical cancer cases over time Relative changes of attended cervical cancer cases before and during the COVID-19 pandemic. A clear drop in the diagnosed cases in 2020 (-19%), followed by an increase in 2022 (+36%), both compared to cases diagnosed in 2019.

This study included records from 797 women with confirmed histopathological diagnoses of cervical cancer between 2019 and 2023. Table 1 summarizes the baseline characteristics of the patients analyzed in this study. Briefly, the median age at cervical cancer diagnosis was 52 years (43-63 years). The median TDT was three months (two to five months). Most patients were diagnosed with locally advanced or metastatic stages. The number of cervical cancer cases diagnosed at our hospital decreased by 19.48% in 2020 compared to the pre-pandemic period. It then increased by 36.3% in 2022 (Figure 1). The most prevalent comorbidities were diabetes and hypertension.

**Table 1:** Demographics of patients with cervical cancer ^1 ^Changes related to the 2019 new cases. Summary of the general characteristics of patients, including obstetric history, cervical diagnostic history, and the number of cases attended by year.

Variable	Median (interquartile range) or n (%)
Obstetric history	
Pregnancies, median (IR)	3 (2 to 4)
Vaginal birth	2 (1 to 4)
Caesarean section	0 (0 to 1)
Abortions	0 (0 to 1)
Age of first sexual intercourse, years	18 (16 to 20)
Number of sexual partners	2 (1 to 3)
Nutritional status n (%)	
Malnutrition	8 (1.0)
Eutrophic	254 (31.9)
Overweight	293 (36.8)
Obesity	223 (28)
Class I	162 (73.6)
Class II	47 (21.4)
Class III	11 (5.0)
Comorbidities	
Diabetes (DM)	227 (28.4)
Hypertension (HT)	197 (24.7)
DM + HT	92 (11.5)
Rheumatoid arthritis	27 (3.4)
Systemic lupus erythematosus	14 (1.8)
Hypothyroidism	12 (1.6)
Chronic kidney failure	7 (0.9)
Smoking	200 (25.1)
Proven HPV infection	527 (65.2)
Age at diagnosis, years	52 (43-63)
Time from diagnosis to treatment, months	3 (2 to 5.0)
Time from screening to treatment, years	1 (0-3)
Clinical stage at diagnosis	
Early	152 (19.1)
Locally advanced	540 (67.8)
Metastatic	105 (13.2)
Year of diagnosis	
2019	154 (19.3)
2020	124 (15.6)
2021	146 (18.3)
2022	210 (26.3)
2023	163 (20.5
Proportion of diagnosed cases by year compared to pre-pandemic cases (2019)^1^	
2019	Reference
2020	-19.48%
2021	-5.19%
2022	+36.36
2023	+5.84%

**Figure 2:**
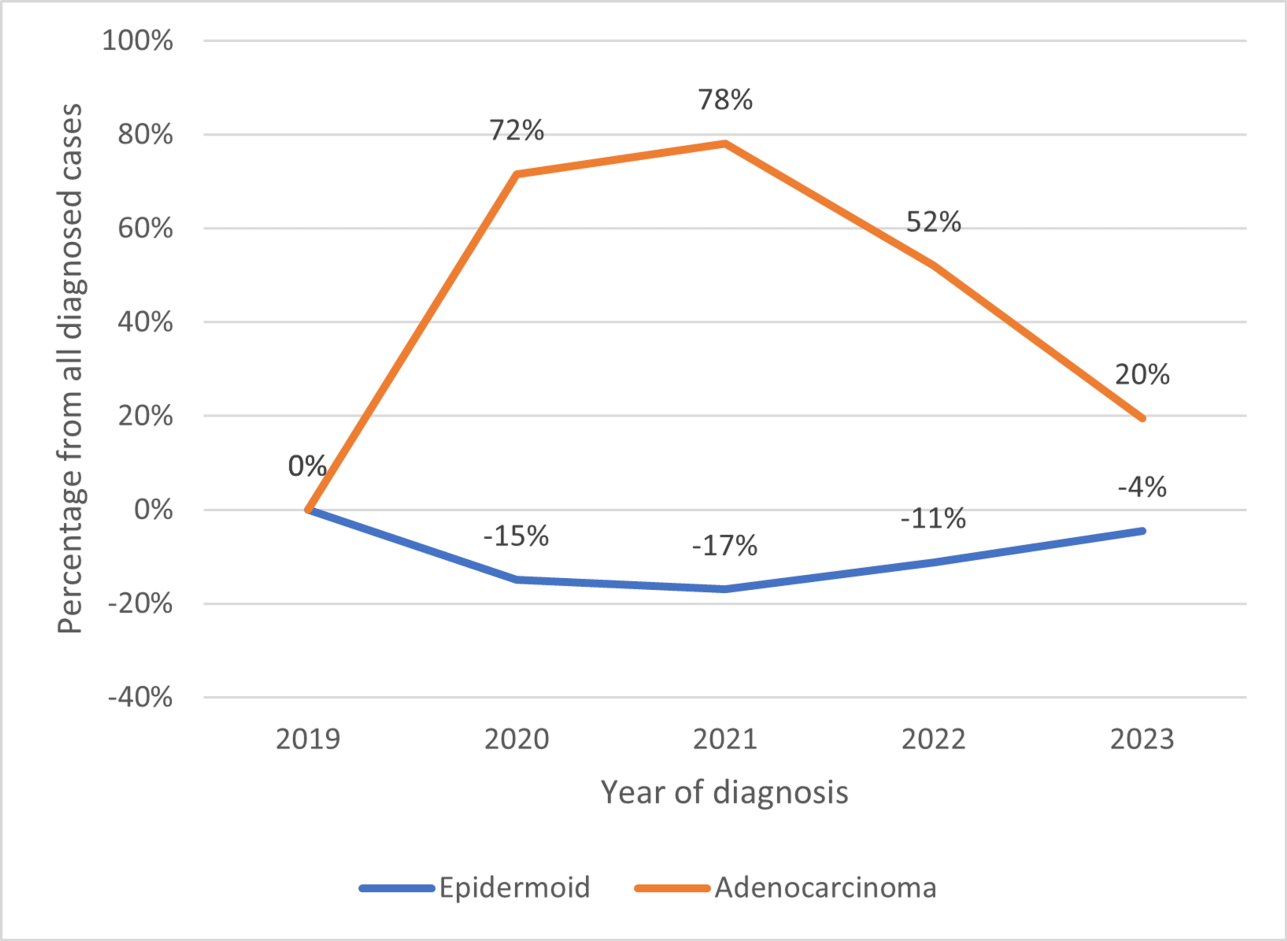
Change in hystological subtype frequencies over time Adenocarcinoma (orange line) cases increased, while epidermoid cases (blue) decreased

Table 2 and Figure 2 show changes in the proportions of histological types of cervical cancer over time. We compared epidermoid, adenocarcinoma, and neuroendocrine subtypes. Significant changes in the proportion of histological subtypes were observed between 2019 and 2020-2022. There was a relative increase in adenocarcinomas at the expense of epidermoid cancers. The rarest types (neuroendocrine and any lymphoid or mesenteric case) remained unchanged over time.

**Table 2:** Histological type of cervical cancer by year of diagnosis Histological cervical cancer types diagnosed each year and significant differences from 2019 frequencies using Chi-squared tests. Notable changes were observed between 2019 and 2022 in the proportions of epidermoid and adenocarcinoma

Histological type	Diagnostic year					Chi-squared value	p-value
	2019	2020	2021	2022	2023		
	n = 154 n (%), reference	n= 124 n (%), Chi-squared value (p)	n=146 n (%), Chi-squared value (p)	n= 210 n (%), Chi-squared value (p)	n=163 n (%), Chi-squared value (p)	11.2	0.19
Epidermoid	127 (82.5)	87 (70.2), 5.87 (0.15)	100 (68.5), 7.94 (0.005)	154 (73.3), 4.04 ( 0.040)	128 (78.8), 0.781 (0.377)		
Adenocarcinoma	26 (16.9)	36 (29.0), 5.85 (0.16)	44 (30.1), 10.52 (0.032)	54 (25.7), 4.21 (0.044)	33(20.2), 0.591 (0.442)		
Neuroendocrine	1(0.6)	1 (0.8), 0.024 (0.878)	2 (1.4), 0.527 (0.971)	2 (1.0), 0.100 (0.752)	2 (1.2), 0.781 (0.377)		

**Figure 3:**
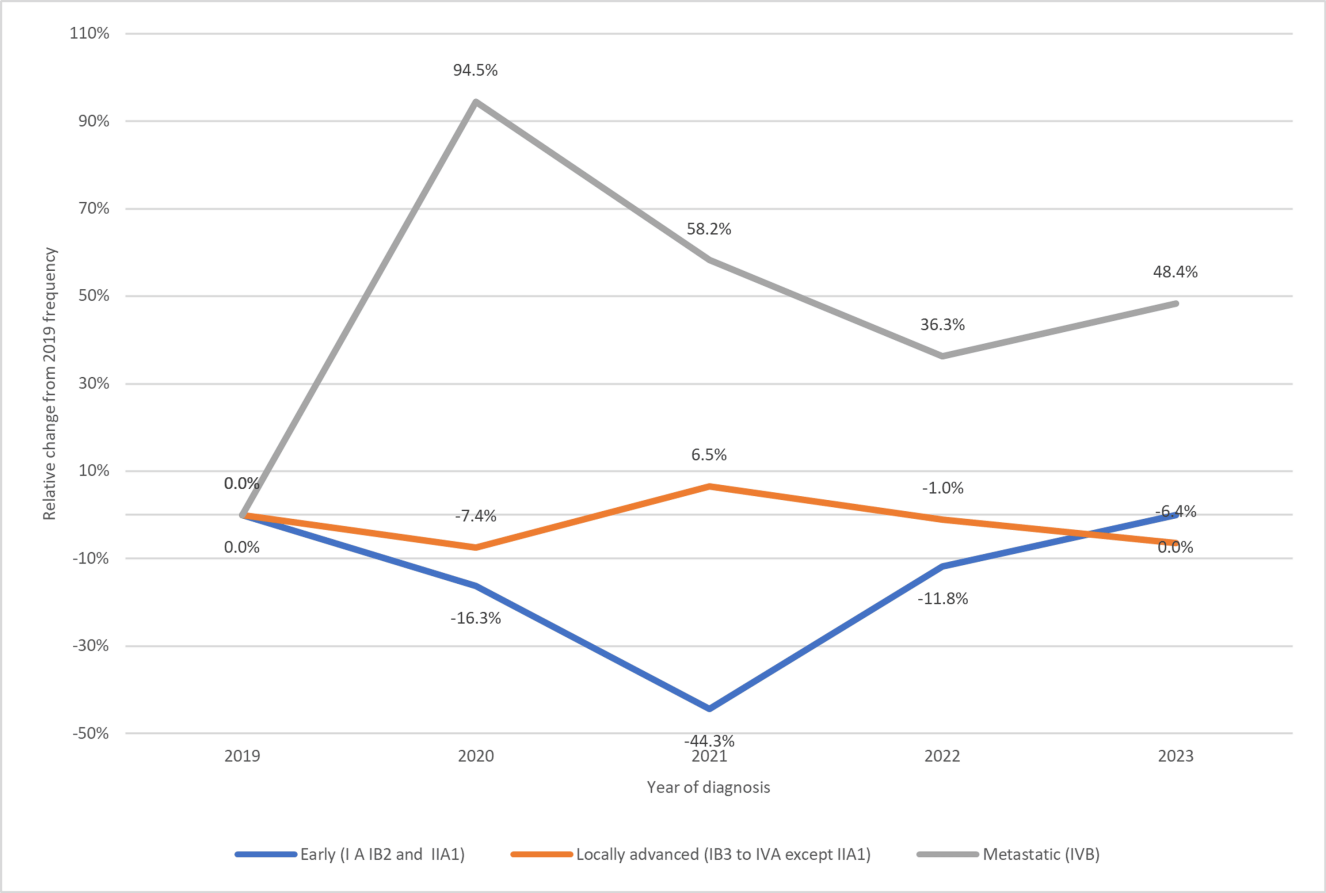
Change in clinical stage at diagnosis over time A relative increase in metastatic cases (gray line) was observed in 2020 (+94.5%), followed by decreases in early (blue line) and locally advanced stages (orange line). A relative reduction of patients diagnosed in early stages was more evident in 2021. Screening programs aim to detect early-stage cases.

Table 3 and Figure 3 compare the clinical stage, TDT, TST, and therapy modalities over time. There was a significant decrease in the early stages, with an increase in the metastatic stage in 2020. The locally advanced stage did not change over time. Specifically, in 2020 and 2021, the time gap from screening to diagnosis doubled. Fewer patients received surgery as their first treatment, with radiotherapy replacing it. Finally, in patients with cervical cancer, obesity and overweight were more prevalent during the pandemic.

**Table 3:** Clinical stage of cervical cancer and treatment at diagnosis *Chi-squared test, ** Kruskal-Wallis test, *** U Man-Whitney test Changes in clinical stages and other diagnostic characteristics over time. In the final column, a global comparison is performed between every year, and in each cell, the specific characteristic is compared with the 2019 frequency.

Variable	Year of diagnosis					Test result	p-value
	2019	2020	2021	2022	2023		
Clinical stage	n=154 n (%)	n= 124 n (%), test result (p)	n= 146 n (%), test result (p)	n= 210 n (%), test result (p)	n= 163 n (%), test result (p)		
Early (IA to (FT1) IB2 and IIA1)	34 (22.1)	23 (18.5), 0.525 (0.469)*	18 (12.3), 4.97 (0.026)*	41 (19.5), 0.357 (0.552)*	36 (22.1), 0.000 (0.999)*	10.47	0.233*
Locally advanced (IB3 to IVA except IIA1)	106 (68.8)	79 (63.7), 0.809 (0.368)*	107 (73.3), 0.723 (0.395)*	143 (68.1),0.022 (0.881)*	105 (64.4), 0.693 (0.405)*		
Metastatic (IVB)	14 (9.1)	22 (17.7), 4.56 (0.033)*	21 (14.4), 2.037 (0.153)*	26 (12.4), 0.983 (0.321)*	22 (13.5), 1.52 (0.217)*		
Time from diagnosis to treatment (TDT), months	3 (2-4)	3 (3-4), 0.004 (0.950)***	4 (2-4), 0.429 (0.512)***	4 (2-5) 1.728 (0.189)***	3 (2-5), 0.094 (0.759)***	3.049	0.550**
Time from screening to treatment (TST), years	1 (0-3)	2 (0-4), 4.987 (0.026)***	2 (1-4) 15.7483 (0.0001)***	1 (0-3), 0.001 (0.975)***	1 (0-3), 0.655 (0.418)***	24.47	< .001>
Treatment groups						8.473	0.389*
Surgery (alone or in combination with other treatments)	38 (29)	36 (32.7)	32 (26.4),	54 (32)	45 (36.6)		
Radiotherapy (alone or in combination with other treatments, except surgery)	88 (67.2)	66 (60)	81 (66.9)	111 (65.7)	71 (57.7)		
Palliative (any kind)	5 (3.8)	8 (7.3)	8 (6.6)	4 (2.4)	7 (5.7)		
Nutrition state							0.222*
Malnutrition and Eutrophic	63 (41.4)	40 (33.6)	44 (31.4)	67 (32.7)	48 (29.6)	5.705	
Overweight and obesity	89 (58.6)	79 (66.4), 1.78 (0.187)*	96 (68.6), 3.15 (0.076)*	138 (67.3), 2.89 (0.089)*	114 (70.4), 4.49 (0.029)*		

The comparison of clinical stage at diagnosis, TDT, TST, treatment modality, and BMI by the pandemic phase (pre-pandemic, early pandemic, and late pandemic) is shown in Table 4. Notably, during the early pandemic period, we observed more cases with metastasis, and there was a significant increase in TST and BMI compared with the pre-pandemic phase. In the late pandemic period, we observed a return to the pre-pandemic state of these characteristics, except for nutritional status.

**Table 4:** Clinical stage, treatment, and nutritional status in the three pandemic phases *Chi-squared test, ** Kruskal-Wallis test, *** U Mann-Whitney test Changes in clinical stage at diagnosis, TDT, TST, treatment modality, and BMI over time, considering three distinct phases: pre-pandemic (2019), early pandemic (2020 – 2021), and late pandemic (2022 – 2023). In the final column, a global comparison is performed for each period, and in each cell, the specific characteristic is compared with pre-pandemic frequencies.

Variables	Year of diagnosis			Test result	p-value
	2019 (prepandemic)	Early pandemic 2020-2021	Late pandemic (2022-2023		
Clinical stage	n=154 n (%), REFERENCE	n= 270 n (%), test result (p)	n= 373 n (%), test result (p)		
Early (IA to (FT1) IB2 and IIA1)	34 (22.1)	41 (15.2),3.2 (0.049)*	77 (20.6), 0.135 (0.71)*	7.045	0.134*
Locally advanced (IB3 to IVA except IIA1)	106 (68.8)	186 (68.9), 0.00 (0.99)*	248 (66.5), 0.271 (0.602)*		
Metastatic (IVB)	14 (9.1)	43 (15.9), 3.93 (0.047)*	48 (12.9), 1.48, (0.221)*		
Time from diagnosis to treatment (TDT), months	3 (2-4)	4 (2-4), -0.363 (0.717)***	3 (2-5), -0.687 (0.434)***	0.461	0.794**
Time from screening to treatment (TST), years	1 (0-3)	2 (1-4), -3.69 (0.0001)***	1 (0-3). -0.403 (0.687)***	21.15	0.0001**
Treatment groups. Test vs prepandemic	Reference	1.560 (0.458)*	1.003 (0.606)*	4.45	0.347*
Surgery (alone or in combination with other treatments)	38 (29)	68 (29.4)	99 (33.9)		
Radiotherapy (alone or in combination with other treatments, except surgery)	88 (67.2)	66 (60)	81 (66.9)		
Palliative (any kind)	5 (3.8)	16 (6.9)	11 (3.8)		
Nutrition status. test pre vs. pandemic	Reference	8.25 (0.041)	6.73, (0.081)	11.95	0.063
Malnutrition and Eutrophic	63 (41.4)	84 (32.4)	115 (31.3)		
Overweight and obesity	89 (58.6)	175 (67.5),	252 (68.7),		

## Discussion

The COVID-19 pandemic negatively impacted the screening of cervical cancer in Mexico [1]; the effect of this missed opportunity for diagnosis is yet to be known. In the present study, we found that the clinical stage at diagnosis was more advanced after the COVID-19 pandemic than in the pre-COVID-19 period, but we cannot conclude whether this was due to a lack of screening in high-risk population groups. 

This finding is aligned with reports from other countries. Bonadio and cols. In Brazil, it was found that cervical cancer was diagnosed in advanced stages after COVID-19. Locally advanced disease (stages III-IVA) increased from 43.3% before COVID-19 to 56.8% one year after the pandemic [6]. It is important to say that the Brazilian screening program for cervical cancer is aimed at sexually active women between 25 and 64 years, recommending a Pap test every three years; both characteristics that are very similar to the policies in our country. As in the Brazilian experience, the major impact in our study occurred during the first year of the pandemic.

The clinical stage at diagnosis of cervical cancer is a major determinant of the treatment plan for the patients. It is assumed that changes in treatment patterns may reflect the proportions of the stages at which the cancer is diagnosed. A retrospective study that compared the cervical cancers diagnosed before the pandemic to the first 24 months of the SARS-CoV-2 pandemic using a multicentric hospital database in Romania reported that the likelihood of cervical cancer requiring radiotherapy increased by 20%, while the possibility of disease progression in advanced FIGO stages was 3.39 (IC 95% 2.06 - 4.21, p<0.001), the authors atribued this changes to the fact that COVID-19 pandemic postposed the diagnosis and treatment start, leading to missed appointments, which predicted cancer progression (HR 2.51, IC 95%) [7]. Another study from Romania reported that Pap screening decreased by 75.5% during COVID-19, a four-week delay in accessing cancer care, an increase in cervical cancer stage III, and a significant decrease in stage I [8]. Although the policies at the Mexican Institute of Social Security ensured the continuity of cancer care during the pandemic, including the availability of surgical services, our results suggest that treatment patterns for cervical cancer changed, with more patients receiving radiotherapy without surgery during the first pandemic year.

The posibility to have a cervical cancer diagnosis, is determined by two means, the screening of disease in early and non clinical evident phases, or because the disease have non deniable expression, in the Brazilian retrospective cohort of 29,796 new cancer cases (all types) showed a drop of 25% of new cancer diagnosis between 2018 and 2020, and a significative increase of 22% by 2021, clinical advanced stages decreased from 17.8% in 2018 to 15.2% in 2020. On the other hand, the time from diagnosis to treatment increased in Brazil for cervical/uterine cancer from 75 to 55 days [9]. This could be part of national changes, not only attributable to health systems, for example, the availability of workers, transportation, and costs.

In our study, the TST for cervical cancer increased during the pandemic. We hypothesize that a delay in seeking medical care from the patients due to the fear of leaving their homes or difficulties in referral to the oncologists from the overwhelmed primary care physicians had a negative impact on the clinical stage at diagnosis of cervical cancer. A study in Belgium saw a shift in the clinical stage of cervical cancer from early to late-stage tumors, presumably because of the priority in the care of symptomatic diseases [10]. Another study conducted in Europe observed a trend in migration from stage II to stage III cervical cancer, while no large tumors were reported [11].

The missuse of -19%, during the first year of pandemic, and the overuse of it in about 38%, was seen in this study, and in England, cervical cancer diagnoses dropped during the pandemic, and they also estimate an overuse of cancer services in the next three years to attend to all the straggler patients for about 919 cases in the country [12], in this three year period, we have seen that the cancer service is still above the prepandemic era (+6%).

In this study, the locally advanced clinical stage did not change over time, but in Japan, this stage increased during the pandemic [13]. This difference cannot be easily interpreted, but the Japanese study used its own staging system, in which N1 or N2 (in the TNM system) corresponds to the IIIC stage, leading to more locally advanced cases being detected.

The impact of screening underuse is a concern for all countries. A predictive model based on the health system of Canada predicted 2% of more cancer deaths from 2020 to 2030, with the most significant impact expected for breast cancer (+5.5%), and cervical cancer (2.2%) [14]. In the same way, cervical cancer diagnoses dropped during the pandemic in England, and they also estimate an overuse of cancer services in the next three years to attend to all the straggler patients for about 919 cases in the country [12]. These reports are consistent with the 6% increase in cancer services use that we have seen, compared to the pre-pandemic era.

In Mexico, the lockdown was mandatory for all citizens, and the BMI in patients diagnosed with cervical cancer is higher after the COVID-19 pandemic, as seen in Canada [15]. The radical change in physical activity, junk food consumption, and mental health problems has been associated with the significant increase in BMI during the pandemic. In countries with mandatory stay-at-home orders, like some states in the USA, the effect on population BMI was worse [16].

After the early pandemic, the Mexican Institute of Social Security has significantly strengthened essential health services, including cervical cancer screening, which has exceeded pre-pandemic levels by 112% [17]. We hope this effort can be maintained even during health crises such as COVID-19, and that, if the association we have observed is causal, it leads to identifying non-symptomatic patients and giving them the opportunity to be diagnosed at early stages.

Limitations relevant to this study include the possibility that cancer patients attended other units during 2020; this is low, as during the pandemic, almost all other units were converted to COVID-19 care, and cancer patients were reoriented to our unit during 2020 and 2021. The clinical stage at diagnosis may have been erroneously recorded in the file, but oncologists in this group revised each case to confirm the clinical stage, using the reports in the file. The time from screening measurement may have memory bias, but it could be interpreted as a systematic error, since every case is treated equally for this variable, and we did not control for this time in the analysis by clinical stage. Since this study has a retrospective design, the clinical files may contain errors or omissions that cannot be corrected. In these cases, clinical stages were taken directly from the file.

## Conclusions

A relative increase of clinically evident cases of cervical cancer, such as metastatic stage, during the first years of the pandemic or "early pandemic", may be related to the observed increase in the time gap from screening to diagnosis observed in this study, and the clinical stages that are clinical silent, like early stage and epidermoid types, were diagnosed in a minor proportion and may be a challenge to the institute during the following years.

Although this is a single-center observation, it is important to observe the association with the pandemic, and we propose that, at long last, screening programs should be a priority, even in emergencies like COVID-19, where communication and population sensitization must be maintained during and after such emergencies, in order to attend to the silent and early stages of this disease.

We propose that, an unexpected relative increase of metastatic cases in the third level of health attention, could be associated to the use of the screening program int he first level, and this must alert the healthcare system to perform every kind of strategy to ensure every woman has the chance to have a diagnosis of cervical cancer in preclinical lesions (CIN) or early stages, to improve the prognosis, and reduce the costs associated with attention for these persons.
